# Reversible thermally induced spin crossover in the myoglobin–nitrito adduct directly monitored by resonance Raman spectroscopy†

**DOI:** 10.1039/d3ra00225j

**Published:** 2023-03-20

**Authors:** Vasiliki K. Valianti, Charalampos Tselios, Eftychia Pinakoulaki

**Affiliations:** a Department of Chemistry, University of Cyprus 2109 Aglantzia Cyprus effiep@ucy.ac.cy; b Department of Chemical Engineering, Cyprus University of Technology Lemesos Cyprus

## Abstract

Myoglobin has been demonstrated to function as a nitrite reductase to produce nitric oxide during hypoxia. One of the most intriguing aspects of the myoglobin/nitrite interactions revealed so far is the unusual O-binding mode of nitrite to the ferric heme iron, although conflicting data have been reported for the electronic structure of this complex also raising the possibility of linkage isomerism. In this work, we applied resonance Raman spectroscopy in a temperature-dependent approach to investigate the binding of nitrite to ferric myoglobin and the properties of the formed adduct from ambient to low temperatures (293 K to 153 K). At ambient temperature the high spin state of the ferric heme Fe–O–N=O species is present and upon decreasing the temperature the low spin state is populated, demonstrating that a thermally-induced spin crossover phenomenon takes place analogous to what has been observed in many transition metal complexes. The observed spin crossover is fully reversible and is not due to linkage isomerism, since the O-binding mode is retained upon the spin transition. The role of the heme pocket environment in controlling the nitrite binding mode and spin transition is discussed.

## Abstract 2

At ambient temperature the high spin state of the heme Fe–O–N=O myoglobin species is dominant and upon decreasing the temperature the low spin state is populated, demonstrating that a thermally-induced spin crossover phenomenon takes place.

## Graphical abstract

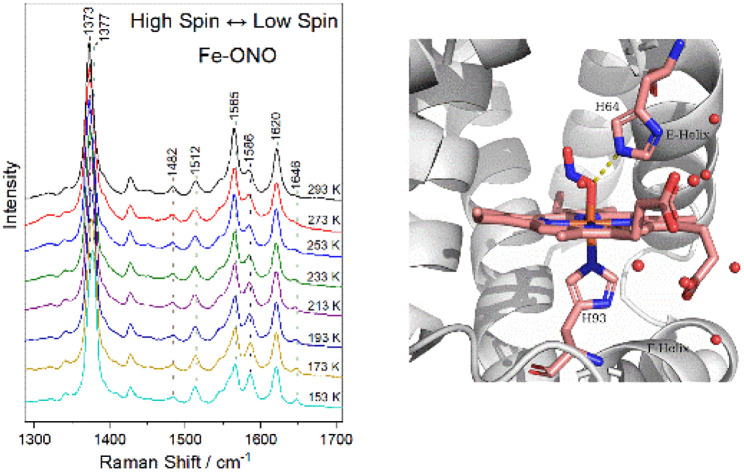

## Introduction

Heme-proteins achieve a wide and divergent range of physiological functions, with the prosthetic group of the Fe derivative of protoporphyrin IX being the vital key for their activities.^1–3^ Among the most studied heme-proteins are vertebrate myoglobin (Mb) and hemoglobin (Hb), well-known for their roles in oxygen storage and transport. During the last few years, new functions of these proteins have emerged including their ability to function as nitrite reductases (NiRs) in mammals, transforming NO_2_^−^ to NO and thus supporting NO signalling during metabolic stress and hypoxic conditions.^4–7^ The reaction pathway involves NO_2_^−^ binding to the heme Fe and thus, elucidating the structural properties and electronic configuration of various heme–nitrite adducts is crucial for understanding the biochemical reaction mechanism.

Most studies on the reactions of NO_2_^−^ with metalloproteins have been focusing on denitrification enzymes (Cu-NiRs and cytochrome *cd*_1_-NiRs) and more lately on the heme-globins, revealing interesting and diverse aspects of NO_2_^−^ coordination and reduction chemistry. Nitrite is a bidentate ligand that can coordinate to metal centers in a bidentate motif as observed in Cu-NiRs, or *via* the N-atom (nitro-, N-binding mode) as in cytochrome *cd*_1_ NiRs, or less commonly *via* one of its O-atoms (nitrito-, O-binding mode).^8–14^ Linkage isomerism between the nitrito- and nitro-binding modes in metal complexes is possible and has been suggested to play important role in biochemical processes.^15–19^ X-ray crystallographic studies revealed the unusual O-binding mode in ferric Mb and Hb, providing the first examples of this coordination in metalloproteins and this mode was also retained upon photoreduction of the heme Fe in Mb.^11,12,14^ However, EPR spectroscopy and DFT studies raised the possibility of nitrito/nitro linkage isomerism in solution for Mb and Hb.^20^ Moreover, conflicting data have been reported for the electronic configuration of the ferric heme Fe–nitrito (Fe–ONO) adduct. The ferric heme Fe–ONO adduct has been assigned to low spin (LS) or high spin (HS) or LS/HS equilibrium states by different research groups that employed various spectroscopic methods including cryo EPR, room temperature resonance Raman and MCD spectroscopies as well as theoretical calculations.^20–25^

The spin state of heme complexes is closely related to their reactivity and thus important in defining the course of biochemical reactions. In fact, one should consider that ligand/substrate binding to the heme Fe and/or electron transfer reactions are frequently accompanied by spin transitions that control heme-protein functions and mechanisms of enzymatic reactions.^1–3^ In addition, external factors including temperature, light, pressure, electromagnetic fields as well as properties such as ligand type, coordination geometry and secondary bonding interactions can also give rise to spin crossover (SCO) phenomena in transition metal complexes and heme proteins. SCO phenomena are being studied in enzymes as they play vital roles in biochemical reactions, as well as in synthetic heme and non-heme systems due to their potential applications in nanotechnology, molecular electronics and spintronics.^26–33^

Resonance Raman spectroscopy is a valuable tool for detection of SCO phenomena in heme proteins and we have previously utilized it to report an unusual ligand-concentration dependent spin transition in the green pigment of Mb, namely the ferric heme nitrito/2-nitrovinyl species.^23^ The resonance Raman spectra of heme proteins comprise of multiple vibrational modes that are sensitive to oxidation, spin and coordination of the heme Fe center, while isotopic substitution of the ligand allows the observation of the binding mode to the heme Fe center.^23,34,35^ In this work, we employed resonance Raman spectroscopy in a temperature-dependent approach to address if temperature can induce the formation of NO_2_^−^ linkage isomers and/or can affect the electronic configuration of the NO_2_^−^ adducts of ferric Mb. The temperature-dependent (293 K to 153 K) resonance Raman approach enabled us to identify that at ambient temperature the HS state of the ferric heme Fe–ONO species exists and upon step-wise decrease of the temperature the LS state is gradually populated. The temperature-dependent SCO we observe is not due to linkage isomerism, since only the nitrito-binding mode is detected at low temperatures similar to what has been observed at ambient temperature.^22,23^ The thermally induced SCO in ferric heme Fe–ONO Mb is gradual and fully reversible, demonstrating a thermal equilibrium of the HS and LS states.

## Experimental

Lyophilized equine myoglobin (95–100%) and chemicals were purchased from Sigma-Aldrich and were of analytical grade unless otherwise stated. The isotopically labeled Na^15^N^16^O_2_ (98% ^15^N, 95% CP) and Na^15^N^18^O_2_ (98% ^15^N, 90% ^18^O, 95% CP) were also obtained from Sigma-Aldrich. Stock solutions of metmyoglobin were prepared by dissolving lyophilized Mb powder in 50 mM of sodium phosphate at pH 7.4. The Mb–nitrite adducts were prepared by mixing metmyoglobin (metMb) with sodium nitrite at 1 : 1000 ratio at pH 7.4. The samples were left to react for 15 min before the onset of data collection.

Raman data were collected by Horiba Scientific LabRAM spectrograph equipped with a CCD detector and an Olympus BX41 microscope. An Ondax SureLock LM-405 laser with an integrated CleanLine ASE filter was used to provide the excitation wavelength at 405 nm, and a 405 nm Semrock StopLine single-notch filter was used to reject the Rayleigh scattering. The samples were placed in temperature-controlled FTIR600 cell (Linkam Scientific Instruments). The desired temperature was achieved using the T95 and LN95 temperature controllers along with the use of liquid N_2_. The laser power incident on the sample was 20 mW and the total accumulation time for each measurement was 6–18 min. The Raman spectra were calibrated using toluene. OriginPro 2021 software was used for spectra processing and analysis.

## Results and discussion

**Fig. 1:**
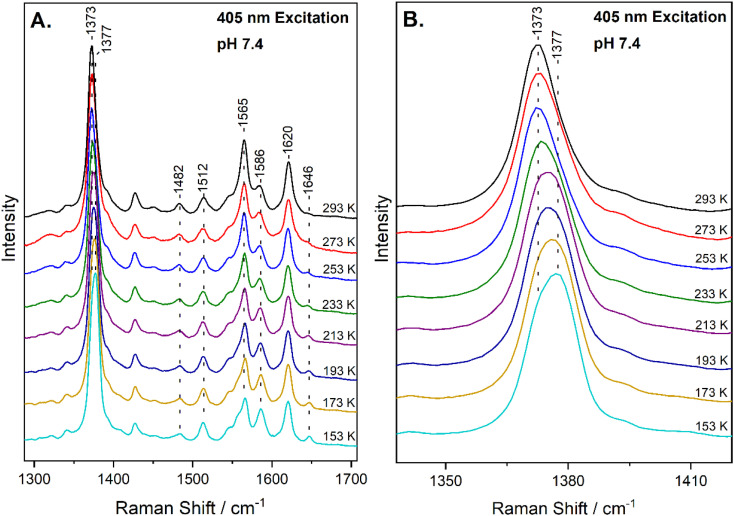
(A) High-frequency resonance Raman spectra of the ferric Mb–nitrite adduct at pH 7.4, obtained at 293 K, 273 K, 253 K, 233 K, 213 K, 193 K, 173 K and 153 K, as indicated in the figure, with 405 nm excitation. (B) Enlarged view of the corresponding spectra in the *ν*_4_ region.

The high-frequency region of the resonance Raman spectra of heme-proteins (1300–1700 cm^−1^) has several modes that are sensitive to the oxidation (*ν*_4_), coordination (*ν*_3_) and spin state (*ν*_2_, *ν*_10_) of the heme Fe. The spectrum of metMb at ambient temperature includes vibrations that are characteristic of six-coordinate HS (6cHS) heme at 1371 cm^−1^ (*ν*_4_), 1482 cm^−1^ (*ν*_3_) and 1563 cm^−1^ (*ν*_2_).^22,35^ The resonance Raman spectrum of the reaction of metMb with NO_2_^−^ at 293 K, shown in Fig. 1A, reveals a slight shift of the *ν*_4_ to 1373 cm^−1^ and *ν*_2_ to 1565 cm^−1^, in agreement with previously published work in which we characterized the reaction species by resonance Raman spectroscopy and identified it as a 6cHS ferric heme Fe–ONO species at ambient temperature.^22,23^Fig. 1A also contains the resonance Raman spectra of the ferric Mb–nitrite adduct obtained by decreasing the temperature in 20 K steps, down to 153 K. Upon decreasing the temperature, the most readily observable change is the development of a band at 1646 cm^−1^ attributed to the *ν*_10_ of 6cLS species, indicating that a thermally-induced SCO transition occurs.^34^ This is also confirmed by the decrease in the intensity of the *ν*_2_ of at 1565 cm^−1^ (6cHS) and concomitant increase of the 1586 cm^−1^ band that is attributed to the *ν*_2_ mode of 6cLS state. Similar observations are made for the HS and LS components of the *ν*_3_ mode at 1482 cm^−1^ and 1512 cm^−1^, respectively. Moreover, upshift of the *ν*_4_ band is observed as temperature decreases, shifting from 1373 cm^−1^ at 293 K to 1377 cm^−1^ at 153 K. An enlarged view of the *ν*_4_ mode is shown in Fig. 1B, demonstrating the presence of the components at 1373 cm^−1^ and 1377 cm^−1^. Overall, the 6cHS species is detected at room temperature, while the 6cLS species is progressively populated when temperature decreases and even at 153 K the 6cHS species is present along with the 6cLS complex. It is noted that the SCO transition we observe is fully reversible and the 6cLS species is reconverted to the 6cHS state when temperature increases to 293 K. The transition temperature for the spin crossover, *T*_1/2_ = 230 K, was calculated by plotting the 1646 cm^−1^ peak area (*ν*_10_ of the 6cLS species) *versus* temperature and fitting of the data, as shown in Fig. S1 (ESI†).

The next important question to address is if the thermally-induced SCO transition we observe could be due to linkage isomerism. Linkage isomerism, that is the presence of both nitrito and nitro-binding modes which can easily interconvert, was previously suggested by cryo EPR experiments and DFT calculations for Mb and Hb.^20^ The nitrite ion, when N-bonded, is a strong field ligand yielding a 6cLS heme species. Resonance Raman spectroscopy can discriminate between the nitro- and nitrito-binding modes by identification of the substrate-bound vibrations.^22,23,36,37^ We have previously demonstrated that at ambient temperature the ferric HS heme Fe–O–N=O Mb adduct is formed in solution and reported its detailed vibration characterization by detecting and assigning the *ν*(Fe–ONO), *δ*(FeONO), *ν*(N–O) and *ν*(N=O) vibrational frequencies.^23^ Herein, we attempt to characterize the corresponding equilibrium HS/LS species that we detect at low temperature.

**Fig. 2:**
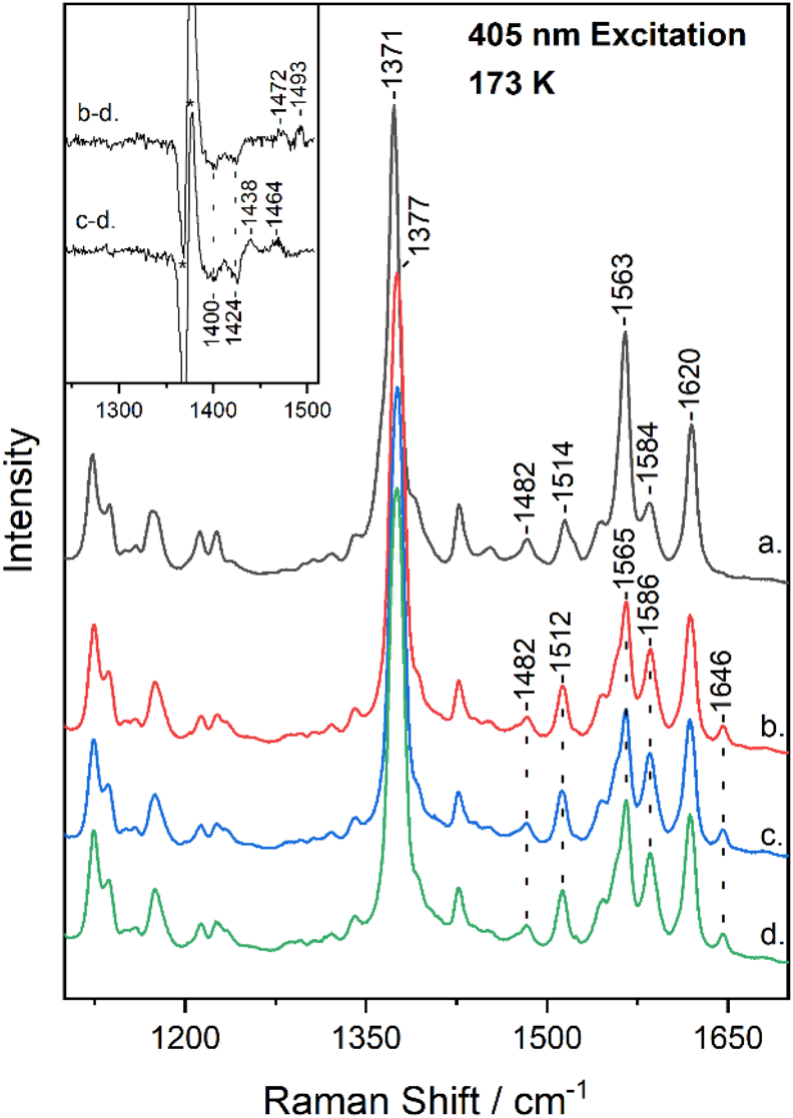
High-frequency resonance Raman spectra of metMb (trace a), and of Mb–^14^N^16^O_2_ (trace b), Mb–^15^N^16^O_2_ (trace c) and Mb–^15^N^18^O_2_ (trace d) adducts at pH 7.4, obtained at 173 K with 405 nm excitation. The difference b − d (Mb–^14^N^16^O_2_*minus* Mb–^15^N^18^O_2_) and c − d (Mb–^15^N^16^O_2_*minus* Mb–^15^N^18^O_2_) spectra are included in the inset. The asterisk denotes intensity differences of the *ν*_4_.

Fig. 2 shows the high-frequency region of the resonance Raman spectrum of metMb (trace a) and after its reaction with ^14^N^16^O_2_^−^ (trace b), ^15^N^16^O_2_^−^ (trace c) and ^15^N^18^O_2_^−^ (trace d) at 173 K. The spectrum of metMb is similar to that obtained at room temperature, excluding the formation of LS metMb species at 173 K.^22^ The spectra of the reactions of metMb with the three NO_2_^−^ isotopes indicate the presence of HS and LS species at 173 K, as it was described for Fig. 1. To identify isotope sensitive vibrations that are extremely weak, difference spectra were calculated and the difference Mb–^14^N^16^O_2_^−^*minus* Mb–^15^N^18^O_2_^−^ (trace b − d) and Mb–^15^N^16^O_2_^−^*minus* Mb–^15^N^18^O_2_^−^ (trace c − d) resonance Raman spectra, are shown in the inset of Fig. 2. The difference b − d spectrum shows two weak peak/trough components at 1472 cm^−1^/1400 cm^−1^ and 1493 cm^−1^/1424 cm^−1^. The difference c − d spectrum, displays peaks at 1438 cm^−1^ and 1464 cm^−1^ and troughs at 1400 cm^−1^ and 1424 cm^−1^. In comparison, the room temperature respective isotope difference spectra displayed a single peak at 1470 cm^−1^ shifting to 1435 cm^−1^ in the ^15^N^16^O_2_^−^ adduct and to 1400 cm^−1^ in the ^15^N^18^O_2_^−^ adduct, which was assigned to the *ν*(N=O) of the HS heme Fe–ONO species.^22,23^ We thus, attribute the 1472 cm^−1^ isotope-sensitive band of the low-temperature spectra to the *ν*(N=O) of the HS heme Fe–ONO species that is similar to the room temperature adduct, while we suggest that the 1493 cm^−1^ band arises from the LS population and its assignment will be discussed below. Heme vinyl nitration does not occur in our experiments, since nitrovinyl bands are not observed.^22,23,38,39^

**Fig. 3:**
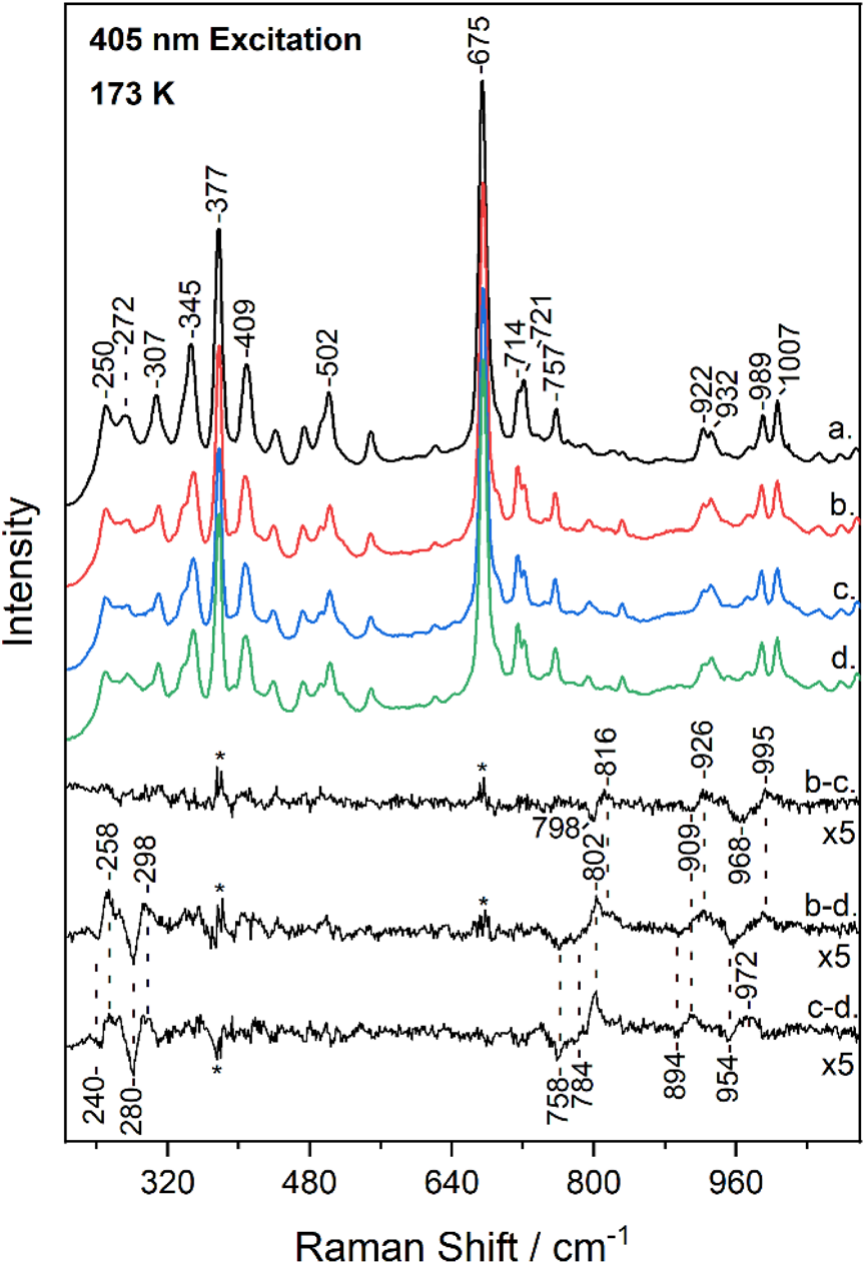
Low-frequency resonance Raman spectra of metMb (trace a), and of Mb–^14^N^16^O_2_ (trace b), Mb–^15^N^16^O_2_ (trace c) and Mb–^15^N^18^O_2_ (trace d) adducts at pH 7.4, obtained at 173 K with 405 nm excitation. The difference b − c (Mb–^14^N^16^O_2_*minus* Mb–^15^N^16^O_2_), b − d (Mb–^14^N^16^O_2_*minus* Mb–^15^N^18^O_2_) and c − d (Mb–^15^N^16^O_2_*minus* Mb–^15^N^18^O_2_) spectra are included in the inset. The asterisk denotes intensity differences in the regions of highest-intensity bands.

Fig. 3 shows the low-frequency (200–1100 cm^−1^) region of the resonance Raman spectrum of metMb (trace a) and after its reaction with ^14^N^16^O_2_^−^ (trace b), ^15^N^16^O_2_^−^ (trace c) and ^15^N^18^O_2_^−^ (trace d), obtained at 173 K. The spectra of metMb as well as those of the reaction of metMb with the NO_2_^−^ isotopes are comparable to the corresponding ambient temperature spectra. Raman bands in low-frequency range include, in addition to vibrations of the porphyrin macrocycle, Fe-ligand motions along the axis normal to the heme unit and thus, substrate isotope difference spectra can provide direct evidence for its binding mode. Therefore, Fig. 3 includes the difference Mb–^14^N^16^O_2_^−^*minus* Mb–^15^N^16^O_2_^−^ (trace b − c), Mb–^14^N^16^O_2_^−^*minus* Mb–^15^N^18^O_2_^−^ (trace b − d) and Mb–^15^N^16^O_2_^−^*minus* Mb–^15^N^18^O_2_^−^ (trace c − d) resonance Raman spectra multiplied by a factor of 5. Trace b − c reveals the N-isotope sensitive modes by the observation of the peak/trough features at 816 cm^−1^/798 cm^−1^, 926 cm^−1^/909 cm^−1^ and 995 cm^−1^/968 cm^−1^. The former and later are similar to those observed at room temperature and assigned to the *δ*(FeONO) and *ν*(N=O) of the heme Fe–ONO species.^22,23^ Traces b − d and c − d include modes that are sensitive to oxygen and oxygen/nitrogen isotopic substitutions, respectively. The peak/trough features at 258 cm^−1^/240 cm^−1^ and 298 cm^−1^/280 cm^−1^, which are O-isotope sensitive, closely match those observed at room temperature and assigned to the *ν*(Fe–ONO) of heme Fe–ONO.^23^ In addition, the peak/trough patterns in traces b − d and c − d further support the assignment of the 816 cm^−1^ and 995 cm^−1^ modes to the *δ*(FeONO) and *ν*(N=O) of the heme Fe–ONO. The feature that was not previously observed in the ambient temperature low-frequency resonance Raman data is the mode at 926 cm^−1^ that shifts to 909 cm^−1^ in the ^15^N^16^O_2_^−^ adduct and to 894 cm^−1^ for the ^15^N^18^O_2_^−^. This band is present only in the low-temperature experiments, thus it arises from the LS population.

Taken together, the low- and high-frequency resonance Raman data obtained at low-temperature demonstrate the presence of heme-bound NO_2_^−^ isotope sensitive bands that closely match those reported previously for the room temperature experiments.^23^ This suggests that the structural properties of the HS heme Fe–ONO population remains largely unaffected by the temperature variation. However, there are two additional isotope sensitive bands at 926 cm^−1^ and 1493 cm^−1^ that are detected in the low-temperature experiments and based on their frequencies and isotopic shifts we assign them to the *ν*(N–O) and *ν*(N=O) of the 6cLS heme Fe–O–N=O population. The presence of a 6cLS heme Fe–nitro adduct at low temperature can be excluded based on the vibrational data, since ferric 6cLS heme Fe–nitro adducts demonstrate distinct vibrational modes as shown for the 6cLS heme Fe–nitro adducts of horseradish peroxidase and *Alcaligenes xylosoxidans* cytochrome *c*′ (AXCP).^36,37^ The fact that we have not been able to identify distinct bands for the *ν*(Fe–ONO) and *δ*(FeONO) of the LS heme Fe–ONO species may indicate that these fall within the same range of the HS adduct or are not observed under the 405 nm excitation.

**Fig. 4:**
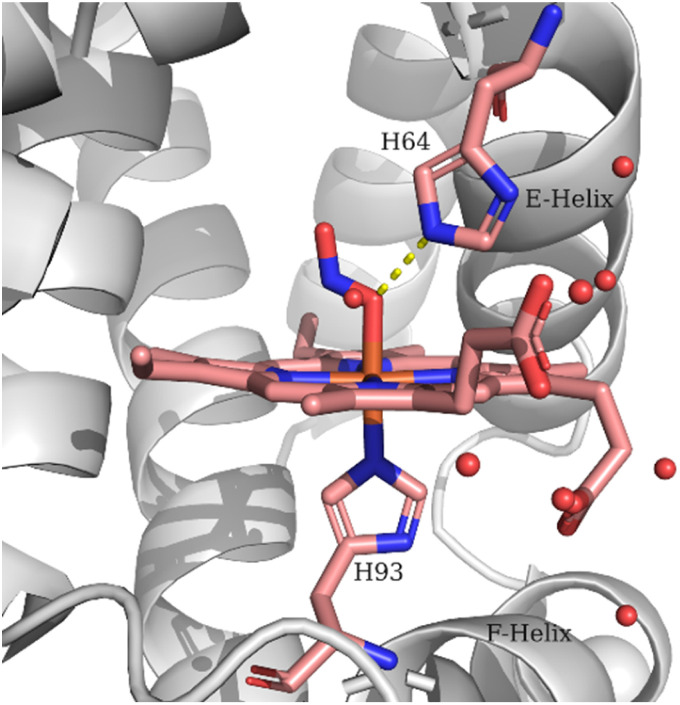
Schematic representation of the Mb heme–ONO species. The structural features are based on PDB code 2FRI.

The observed lower frequency of the *ν*(N=O) and higher frequency of the *ν*(N–O) of the LS heme Fe–ONO compared to the HS heme Fe–ONO species indicate that different hydrogen bonding interactions and/or geometries of the FeONO moiety can exist for the two populations. Evidence for the importance of H-bonding interactions on the NO_2_^−^ coordination mode and geometry have been provided by the crystallographic, spectroscopic and theoretical studies.^11–13,21,22,24,40^ The crystal structure of the ferric Mb–nitrito adduct revealed that the FeONO moiety was in the *trans* conformation and the distal residue His64 was proposed to exert a strong influence in directing the O-binding mode by H-bonding to O1 (Fe–O1–N=O2) as shown in Fig. 4, a hypothesis that was further supported with the observation of the N-binding mode in the crystal structure of the H64V Mb mutant.^11,13^ Moreover, reintroduction of a H-bonding residue into the distal pocket in the H64V/V67R double mutant restored the O-binding mode of nitrite, but in a distorted *cis*-like conformation.^13^ In the case of the nitrite adduct of human tetrameric Hb that also exhibits O-binding, the FeONO moiety was found in the *trans* conformation in the α subunit, while a distorted *cis*-like conformation was observed in the β subunit.^12^ In addition to the role of His64 in directing the O-binding, the role of water molecules in providing H-bonding interactions to the heme bound NO_2_^−^ was suggested by resonance Raman experiments and DFT calculations.^22,24^ The vibrational frequencies derived from the DFT calculations indicated that an upshift of the *ν*(N–O) and a downshift of the *ν*(N=O) would be expected upon H-bonding of the O2 (Fe–O1–N=O2) with H_2_O molecules compared to the non-hydrogen bonded species, which is a motif comparable to that observed for the *ν*(N=O) and *ν*(N–O) of the two populations in the resonance Raman experiments reported herein. In the DFT models the presence of H-bonding from H_2_O molecules also increased the bond length of the Fe-N_His_ (proximal His93), whereas the Fe–O bond exhibited minor changes.^24^

Interestingly, second sphere control of spin state through H-bonding has been recently described in model ferric porphyrin complexes. Examples include five coordinate Fe(iii) octaethyl-tetraarylporphyrin, Fe(iii) porphyrin complexes with covalently attached imidazole or thiolate ligands and axial H_2_O or OH^−^ ligands and heme–peroxo–copper complexes.^30–32^ In the latter study, intramolecular H-bonding interactions were demonstrated to facilitate thermally induced SCO in the synthetic heme–peroxo–copper complexes.^32^ In the case of the temperature-induced SCO of the heme Fe–ONO reported here, the data are consistent with subtle conformational changes in the distal site affecting the hydrogen bonding interactions and/or geometry of the FeONO moiety, although contributions from proximal effects should also be considered. We have previously utilized resonance Raman spectroscopy at ambient temperature to investigate a ligand-concentration dependent spin transition in ferric heme nitrito/2-nitrovinyl species. We suggested that structural rearrangements in the protein binding pocket accompanied by a change in the displacement of the heme Fe along the heme plane without breaking of the heme-ligand bonds were responsible for the spin state transition.^23^ Conformational changes to the E helix in the distal environment, along with the F helix in the proximal site upon removal of excess ligand from the protein cavities were considered as regulating factors.^23,24^ The role of the proximal environment in spin transitions is supported by DFT calculations that showed decrease of the Fe–N_His_ bond length by ∼0.3 Å in the LS state compared to the HS species, while the corresponding Fe–O was less affected.^24^ Bond contraction was also implicated in the temperature induced SCO in neuronal nitric oxide synthase bound with heme-coordinating thioether inhibitors. It was proposed that contraction of the Fe–S thioether bond by ∼0.2 Å below 200 K resulted in an increase in the ligand field strength forcing the pairing of the iron d electrons into the lower energy orbitals and leading to a HS to LS transition.^27^

Finally, we note that the HS state of the heme Fe–ONO species is dominant at ambient temperature and present even at 153 K along with the LS state. The temperature-dependent spectral changes that we observe in the resonance Raman experiments are gradual and reversible, thus indicating that the HS and LS states are in thermal equilibrium. Even though the theoretical studies predict that the energy differences between nitro- and nitrito-isomers are very low for ferric and ferrous porphyrins and thus isomers could interconvert by temperature variations,^19–21,41^ this is not the case for the ferric nitrito–Mb. The protein environment controls the O-binding mode and the thermally induced spin transition is not due to linkage isomerism. Assuming that the O-binding mode is retained upon heme reduction, there would be the advantage of the requirement of a single proton transfer to lead to the formation and release of NO during the catalytic cycle.^41^

## Conclusions

In summary, the present work reports the direct observation of a thermally induced SCO in ferric nitrito–Mb by the application of resonance Raman spectroscopy in a temperature-dependent approach. The observed SCO is not the result of linkage isomerism, since the O-binding mode is retained upon the reversible HS to LS transition. The SCO behaviour of nitrito–Mb underscores the role of the heme pocket in not only orienting ligands/substrates to allow selective reactions, but also in controlling properties such as the spin state that ultimately define reactivity.

## Author contributions

V. K. V. investigation, analysis, writing—original draft preparation; C. T. investigation; E. P. conceptualization, supervision, funding acquisition, writing—review and editing.

## Conflicts of interest

There are no conflicts to declare.

## Supplementary Material

RA-013-D3RA00225J-s001
