# Supplementary material for: Reversible thermally induced spin crossover in the myoglobin–nitrito adduct directly monitored by resonance Raman spectroscopy
Source: RSC Adv. 2023 Mar 20;13(13):9020–5. doi: 10.1039/d3ra00225j (PMC10025812; doi:10.1039/d3ra00225j)
Supplement: RA-013-D3RA00225J-s001 [file RA-013-D3RA00225J-s001.pdf]

Electronic Supplementary Material (ESI) for RSC Advances.
This journal is © The Royal Society of Chemistry 2023

Supplementary Information

Reversible Thermally Induced Spin Crossover in the Myoglobin-Nitrito Adduct Directly Monitored by Resonance Raman Spectroscopy

Vasiliki K. Valianti,[a] Charalampos Tselios [a,b] and Eftychia Pinakoulaki *[a]

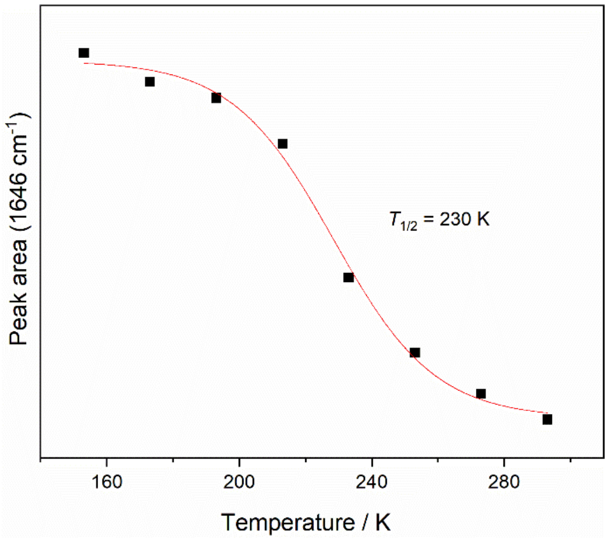

Figure S1: Plot of the 1646 cm^{-1} peak area (ν_{10} of 6cLS species) versus temperature. The sigmoidal fitting of the data indicates that spin crossover occurs with a transition temperature $T_{1/2}$ = 230 K.
